# Investigation of patients’ understanding of fibromyalgia: Results from an online qualitative survey

**DOI:** 10.1016/j.pec.2024.108156

**Published:** 2024-01-17

**Authors:** Lora L. Black, William R. Black, Andrea Chadwick, Jennifer L. Christofferson, Hannah Katz, Monica Kragenbrink

**Affiliations:** a Department of Psychiatry & Behavioral Health, The Ohio State University Wexner Medical Center, 1670 Upham Drive, Columbus, OH 43210, USA; b Center for Biobehavioral Health, The Abigail Wexner Research Institute at Nationwide Children’s Hospital, 575 Children’s Roadd, Columbus, OH 43215, USA; c Department of Pediatrics, The Ohio State University College of Medicine, 370 W. 9th Avenue, Columbus, OH 43210, USA; d Department of Anesthesiology, The University of Kansas School of Medicine, 3901 Rainbow Boulevard, Kansas City, KS 66160, USA; e Center for Children’s Healthy Lifestyles & Nutrition, Children’s Mercy Hospital, 610 E 22nd Street, Kansas City, MO 64108, USA; f Clinical Child Psychology Doctoral Program, University of Kansas College of Liberal Arts and Sciences, 1000 Sunnyside Avenue, Lawrence, KS 66045, USA; g Department of Psychiatry & Behavioral Sciences, The University of Kansas School of Medicine, 3901 Rainbow Boulevard, Kansas City, KS 66160, USA

**Keywords:** Fibromyalgia, Patient understanding, Qualitative

## Abstract

**Objective:**

An important component to effective fibromyalgia treatment is patient education about the condition. While previous educational interventions have been developed, these have not incorporated the lived experiences of patients and may not address common misunderstandings among those who may benefit from these interventions. This study aimed to explore understanding, confusion, and gaps in knowledge about fibromyalgia among those who report a fibromyalgia diagnosis.

**Methods:**

Participants were recruited via clinic flyers and the social media page of a chronic pain research laboratory. Participants completed an online survey that assessed their knowledge of fibromyalgia via open-ended questions. Responses were analyzed via thematic analysis to identify, analyze, and report themes.

**Results:**

Thirty-eight participants completed the survey (63% female, ages 18–68). Common themes that arose from patients included being unsure of the cause of their fibromyalgia, frustration and confusion about the random/variable nature of symptoms and flares, feeling that their condition was invisible, and desiring more information on available treatments.

**Conclusion:**

Participants in this study expressed confusion about many aspects of fibromyalgia and a desire for more understanding from others in their life about this condition.

**Practice implications:**

Future interventions would benefit from tailoring fibromyalgia education to the specific knowledge and lived experiences of patients.

## 1. Introduction

Fibromyalgia is a chronic condition characterized by widespread musculoskeletal pain or tenderness that is often accompanied by somatic complaints including fatigue, sleep disturbance, headaches, and cognitive difficulties [1,2]. In addition, it is well-documented that patients with fibromyalgia have higher rates of depression and anxiety compared to healthy controls [3,4] that can further reduce a patient’s quality of life; indeed, persons with fibromyalgia and their family members report lower quality of life than those without a chronic pain condition [5]. The large impact that this condition can have on someone’s physical, psychological, and social functioning coupled with the lack of objective diagnostic tests make it imperative to better understand the subjective experiences of patients with fibromyalgia.

Despite prevalence rates up to 2–8% of the population [1], fibromyalgia is still often underdiagnosed by medical providers and is poorly understood by many patients with the condition [2,6]. Poor patient understanding of their medical condition is associated with worse treatment adherence among those with chronic illnesses [7–9], demonstrating the vital need for providing fibromyalgia education as a core component of treatment in addition to medication management, physical activity, pain-focused psychotherapy, sleep hygiene, and general symptom management [10].

A number of pain education interventions have been created and shown to be effective in increasing knowledge and decreasing pain catastrophizing [10–13] but are significantly limited in two ways. First, these interventions have been developed by pain scientists and healthcare providers with little to no patient input, therefore these interventions may not provide information of greatest relevance and importance to patients as they present in clinic. Second, educational interventions are generally intended to be administered face-to-face. However, there has been increased demand for virtual and self-guided online interventions for chronic pain [14,15]. Given the need for and benefit of personalized content to best reflect individual patient experiences [16,17], it is not clear how well in-person interventions translate to self-guided formats if patient input is not first considered. It is also unclear whether the transition to self-guided formats ultimately affects treatment engagement and effectiveness. Chronic pain education interventions could be improved with a greater recognition of patients’ common questions and misunderstandings about their condition.

Patient understanding and knowledge of fibromyalgia has generally been assessed via close-ended questions or knowledge tests [13]. Unfortunately, this method of close-ended questioning may miss nuanced information that could be critically relevant to patients’ understanding of fibromyalgia. One previous study used qualitative methods to explore patient’s perception of the causes of their fibromyalgia [18] and found that fibromyalgia was attributed to experiencing some assault to the body or ill-health, emotional trauma/distress, or stress from their daily lives. However, that study focused solely on patient’s views regarding the cause of their condition and did not assess other facets of fibromyalgia that could be targeted in educational interventions, such as desired areas for additional education for both themselves and their loved ones. To the authors’ knowledge, the current study is the first to explore understanding, confusion, and gaps in knowledge about fibromyalgia among those who report being diagnosed with the condition.

## 2. Methods

### 2.1. Design

Participants were recruited via flyers located in comprehensive spine center clinics and online via a chronic pain research lab Facebook page directing interested participants to an online survey. The online survey was administered using REDCap electronic data capture tools hosted at the study’s institution [19,20]. Information about the survey was provided and participants were directed to questions after they agreed to take part in the research study. Participants were able to skip questions, partially complete questions, complete the survey at a later time, or withdraw their consent by not submitting their answers. All study procedures were approved by the institution’s IRB. The online mixed-methods survey assessed participant demographics via multiple choice options and knowledge of fibromyalgia via open-ended questions. Specifically, patients were asked about what they thought caused their fibromyalgia, what they thought patients with fibromyalgia wanted to know most about the condition, what confuses them most about fibromyalgia, and what they wanted other people in their life to understand about fibromyalgia. After participants completed study questions, they were directed to a separate survey where they could provide contact information to be compensated $30 via a prepaid debit card (ClinCard). All contact information was collected and stored separately from survey responses and were not linked.

### 2.2. Participants

Eligible participants were those 18 or older who self-reported a previous diagnosis of fibromyalgia. Thirty-eight participants completed the online survey over a three-week period. The mean age was 36.16 years with a range of 18–68 years. The majority of participants were female (63.2%), White (62.2%), married/partnered (50%), and full or part-time employment (76.3%). Participants were asked when they were diagnosed with fibromyalgia through open-ended questions and the answers ranged from less than a year to over 30 years ago.

### 2.3. Analysis

Responses were analyzed via thematic analysis to identify, analyze, and report themes found within the data [21] using Dedoose [22]. Two team members (LLB and WRB) reviewed the open-ended responses and developed codes, which were discussed and agreed upon by team members. These team members (LLB and WRB) then independently categorized responses into the codes. As this study utilized open-ended survey questions rather than traditional qualitative interviews, many of the responses were brief. While some participants provided more elaborate responses, no one response or participant was given more weight during the analysis. Any discrepancies in coding were discussed until consensus was reached. In the event consensus was not reached, a third team member (JLC) reviewed the responses and codes to decide on final coding categorization. All themes were developed from the iterative review of all 38 participants where the two primary coders grouped similar responses together into codes which were then assigned a theme to best represent the group of responses.

## 3. Results

Four broad themes corresponding to patients’ understanding of and uncertainty related to fibromyalgia were identified from the open-ended survey data. These included patient’s beliefs and confusion about what caused their fibromyalgia, the random and unpredictable nature of symptoms, the lack of understanding from others about their condition, and desire for appropriate treatment recommendations. Table 1 and Table 2.

### 3.1. Patients’ beliefs about what caused their fibromyalgia

Many patients reported that they were either unsure or did not know the initial cause of their fibromyalgia and would like to better understand what caused their chronic pain condition.

“I wish I knew.” (Age not reported, Female, White, Married, Retired, Pain started 10 years ago, Diagnosed with fibromyalgia 10 years ago).

“I don’t know however I don’t wish it on anyone.” (Age not reported, Female, White, Married, Employed part-time, Pain started 18 years ago, Diagnosed with fibromyalgia 7 years ago).

Another common response was the belief that emotional trauma/stress caused their fibromyalgia. While some participants provided more general answers of “emotional stress” or “trauma”, others specifically identified the stress of being victims of intimate partner violence as the trigger for their symptoms. Others reported that specific accidents or physical trauma (e.g., “a bad car accident”), preexisting problems with specific parts of their body (e.g., “back problems”, “muscles”, “nerves”), outside influence (e.g., “unknown chemicals”, “food”), or genetics/family inheritance were the cause of their fibromyalgia.

### 3.2. The random and unpredictable nature of symptoms

Several participants expressed confusion about their symptoms, including the seeming random nature of their pain flares, variability of symptoms between different people or within themselves on different days, as well as the unpredictability of these symptoms.

“I used to wonder why some people you would practically blow on [them] and it would hurt [but] with me it wasn’t that way at all.” (68, Female, Black/African American, Divorced, Retired, Pain since childhood, Did not report when diagnosed with fibromyalgia).

“…the flares just happen out of the blue. Some are [worse] than others. There is really no rhyme or reason to it.” (39, Female, White, Single, Not working/disabled, Pain started 10 years ago, Diagnosed with fibromyalgia 10 years ago).

### 3.3. Patients’ feelings that fibromyalgia is an invisible condition that others do not understand

Many participants reported confusion and frustration about how fibromyalgia is an “invisible” condition that makes it hard for others to believe their symptoms are real.

“Most people do not understand how very debilitating this disease is and has been for me. I get looked at as if I am crazy from family and friends on my bad days or when I cancel engagements due to pain.” (40, Female, White, Married, Employed part-time, Pain started 6 years ago, Diagnosed with fibromyalgia 6 years ago).

“Not really confusing just frustrating when no one believes that you hurt and can’t move some days.” (Age not reported, Female, White, Married, Employed part-time, Pain started 18 years ago, Diagnosed with fibromyalgia 7 years ago).

Others reported wanting others to understand that the pain they experience is real and impacts their daily lives.

“[Fibromyalgia is] a real condition. It is very painful, and we go through flares. We are not lazy, we are in actual pain and can’t do everything like a normal person can.” (39, Female, White, Single, Not working/disabled, Pain started 10 years ago, Diagnosed with fibromyalgia 10 years ago).

“I’m NOT making this up. It’s NOT just in my head. That just because I get up and go to work DOES NOT mean I’m not having pain. I function in pain every single day.” (52, Female, White, Single, Employed full-time, Pain started at least 15 years ago, Diagnosed with fibromyalgia 15 years ago).

### 3.4. Patients’ desire for fibromyalgia treatment options and cures

While some participants expressed interest in learning about ways to cure fibromyalgia (e.g., “n*ew advancements in science toward a cure/treatments,” “total prevention/cure*”), others discussed how fibromyalgia is a life-long condition with no cure but that there are treatment options.

“I would like people to understand that there is no cure for fibromyalgia and while some things can make it easier to live with it, I’m in a lot of pain and it will never go away.” (41, Female, White, Partnered, Employed full-time, Pain started 3 years ago, Diagnosed with fibromyalgia 1.5 years ago).

“[Fibromyalgia] is something that cannot be cured but the person can be helped to live with it.” (68, Female, Black/African American, Divorced, Retired, Pain since childhood, Did not report when diagnosed with fibromyalgia).

However, participants still expressed a need to better understand what these specific treatments include:

“…what medications can be of help, what treatments, daily routines or exercises one can do to reduce symptoms and pain.” (41, Female, White, Partnered, Not working/disabled, Pain started 7 years ago, Diagnosed with fibromyalgia 4 years ago).

“The quickest most effective treatment options.” (30, Male, Black/African American, Married, Employed full-time, Pain started 6 months ago, Diagnosed with fibromyalgia 6 months ago).

## 4. Discussion and conclusion

### 4.1. Discussion

There are several well-established pain educational programs with versions developed for both clinicians and patients [12,23]. However, these educational interventions may lack specificity sought by patients with fibromyalgia. Furthermore, educational interventions are also thought to be necessary but insufficient for behavior change [24]. Thus, despite the effectiveness of these educational interventions for increasing knowledge and understanding of fibromyalgia, further development of these interventions can improve their efficacy, especially given the need for patients to actively participate in fibromyalgia treatment [10].

The findings of this study provide a better understanding of fibromyalgia patients’ knowledge and specific concerns about their chronic pain condition. A number of participants pointed to specific instances of physical trauma, previous illness, and psychological stress as the cause of their fibromyalgia. This is consistent with previous research investigating patient understanding of fibromyalgia etiology [18]. However, that study included forced-choice response options and did not allow for patients to indicate that they did not know the cause of their chronic pain condition. Thus, this previous research was unable to capture themes associated with patients’ lack of knowledge. A central theme of this study is that even when queried in an open-ended fashion, many patients were unsure of what caused their fibromyalgia and wanted more information. Some potential causes have been suggested, and numerous studies have shown associations between physical and/or psychological trauma and the development of fibromyalgia [25]. However, this body of work may lack the scientific rigor needed for drawing definitive conclusions regarding distinct causes, triggers, and inciting factors for fibromyalgia [25,26]. It is thus difficult to determine whether patients’ poor understanding of fibromyalgia development is due to gaps in educational interventions or conflicting and confusing messages from the medical community. While educational interventions should acknowledge and discuss extant literature, such as the common experience of trauma among fibromyalgia patients, they should also discuss how our understanding of the condition is still evolving.

Another theme of the current study was the unpredictability of symptoms and the seemingly random nature of flares. This is consistent with the known dynamic nature of chronic pain conditions [27–29]. Further, a number of participants expressed confusion about the range of symptom variability in people with the same diagnosis. This is similar to previous studies showing the wide variety of symptoms patients attribute to fibromyalgia [30,31] and the high number of people searching online about fibromyalgia symptoms [32]. As symptom variability between and within fibromyalgia patients is common, this is likely another important area to be addressed in fibromyalgia education as both a means to validate their own experience, and to caution them against comparing their symptoms to others. Such education may also lay the groundwork for providers to better manage patient expectations for flares as well as the importance of developing different coping skills for different symptoms.

In alignment with previous research [33–36], participants in the current study also reported that fibromyalgia feels like an invisible illness and believe this leads to others doubting the legitimacy of their condition. These experiences of invalidation may impact health outcomes [37] and increase psychological stress that may in turn amplify pain [38]. Patients expressed wanting to be able to make others understand that their condition and pain is “real.” A number of participants in this study also wanted information about fibromyalgia prevention, cures, and treatment options. As the most effective treatment protocols for fibromyalgia focus on symptom management versus total elimination of pain [10,39,40], this is important for providers to address in their work with such patients. However, given the complexity of fibromyalgia and the general concerns of invalidation from both their social supports and the broader community, additional work is needed to address both when and how to provide such information in order to maximize understanding and uptake of information.

This study adds the patient perspective, a much needed but commonly overlooked component, to the overall research about fibromyalgia. However, this study has several limitations that should be addressed. This study utilized a convenience sample of persons who self-reported a diagnosis of fibromyalgia, rather than those whose diagnosis was confirmed via medical chart review but may be illustrative of how the broader population views chronic conditions such as fibromyalgia. However, this sample is largely white, female, employed full-time, and 1–10 years from diagnosis, and may not fully represent the lived experiences of those with fibromyalgia outside of these demographics. The responses from this study may be more representative of those who are relatively early in their fibromyalgia diagnosis (e.g., 1–10 years) and still able to work full-time. Thus, the findings of this study may be more applicable to educational interventions aimed at those seeking treatment earlier in the course of the condition prior to the onset of significant functional deficits. Further, this study analyzed open-ended responses from a qualitative perspective and are likely less detailed than responses gathered from formal qualitative individual interviews or focus groups. While limited, the responses may be able to guide future qualitative and quantitative studies on patient perspectives about fibromyalgia.

### 4.2. Conclusion

Overall, this study highlights the need for better fibromyalgia education, during both formal educational interventions as well as regular clinical encounters, and provides specific educational targets for future educational interventions. Many participants expressed poor understanding regarding multiple aspects of fibromyalgia and were interested in learning more about areas such as etiology and treatment options. This desire for more information provides natural buy-in for educational interventions.

### 4.3. Practice implications

While a significant amount of information can and should be provided at the time of initial diagnosis, fibromyalgia is highly variable between and within patients. As such, this study supports the need for ongoing education as it becomes relevant based upon a patient’s unique clinical presentation and is consistent with current recommendations to continue education throughout follow up treatment [10]. Incorporating findings from the current and other studies that acknowledges patients’ lived experiences with a condition that is often described as confusing and invisible can help to reduce feelings of invalidation and increase patient engagement. Moving forward, our team aims to create education interventions that address these common fibromyalgia experiences and misconceptions by explicitly creating an opportunity for patients to provide their thoughts and experiences with chronic pain and/or specifically highlighting common themes that patients with such conditions report as part of the didactic material. Further, we intend to solicit patient preferences for education interventions (e.g., modality, length, qualities of interventionalist) to better tailor such efforts for maximum patient engagement. We believe that using patient narratives of their fibromyalgia experiences and preferences for intervention delivery can help them to feel heard and understood and is vital for effective education and management of fibromyalgia.

## Figures and Tables

**Table 1:** Participant demographics.

Demographic variable	n	%
Age, mean(range)	36.16(18-68)	
Gender		
Female	24	63.2
Male	14	36.8
Time since diagnosis		
Less than 1 year	2	5.3
1-10 years	21	55.3
11-20 years	4	10.5
More than 20 years	3	7.9
Unknown/not reported	8	21.0
Race/Ethnicity		
Black/African American	14	36.8
White	23	60.5
Hispanic	1	2.6
Relationship status		
Married	15	39.5
Partnered	4	10.5
Single	16	42.1
Divorced	2	5.3
Widowed	1	2.6
Employment status		
Full-time employment	20	52.6
Part-time employment	9	23.7
Retired	3	7.9
Not employed, disabled	3	7.9
Unemployed	2	5.3
Full-time student	1	2.6

**Table 2:** Open-ended survey questions and associated codes.

Question: What do you think is the cause of your fibromyalgia?	Number of responses
Total number of participants’ responses analyzed: 32	
Participant does not know or is unsure what caused their fibromyalgia.	8
Participant references a preexisting condition in a specific body part as the cause of their fibromyalgia.	3
Participant believes that a history of emotional trauma or stress caused their fibromyalgia.	5
Participant believes that a past physical trauma or accident caused their fibromyalgia.	6
Patient believes that an outside influence (e.g., food, chemicals) caused their fibromyalgia	3
Patient believes that their fibromyalgia is a genetic condition that they inherited.	7
**Question: What do you think patients want to know most about fibromyalgia?**	
**Total number of participants’ responses analyzed: 29**	
Participant would like to know more about their symptoms and flares.	2
Participant would like to know more about what caused their fibromyalgia.	6
Participant would like to know how to stop fibromyalgia or cure it.	7
Participant would like to know more about symptom management and/or treatment options, including ways to better cope with the symptoms.	20
**Question: What confuses you the most about your fibromyalgia?**	
**Total number of participants’ responses analyzed: 27**	
Participant is confused about the cause of their fibromyalgia.	8
Participant is confused about their symptoms and flares, including how unpredictable and variable the symptoms can be at times.	11
Participant is confused as to the “invisible” nature of fibromyalgia and how this leads to others either not knowing about the condition or not believing them.	6
**Question: What would you like people in your life to understand about fibromyalgia?**	
**Total number of participants’ responses analyzed: 25**	
Participant would like for others to understand that fibromyalgia is a lifelong illness with no cure.	3
Participant would like for others to know that they are in pain and the pain is real.	11
Participant would like for others to be more understanding of them and their fibromyalgia symptoms.	5
Participant would like for others to understand the severity of their fibromyalgia symptoms.	5
Participant would like for others to better understand the large impact that fibromyalgia symptoms have on their lives.	10

## References

[1] Clauw DJ. Fibromyalgia: a clinical review. JAMA 2014;311:1547–55. 24737367 10.1001/jama.2014.3266

[2] Giral CR, Diaz-Manchay R, Leon-Jimenez F. Fibromyalgia: the underdiagnosed disease of pain. Reumatol/Rheumatol 2021;59:64. 10.5114/reum.2021.103452 PMC7944961 33707799

[3] Borchers AT, Gershwin ME. Fibromyalgia: a critical and comprehensive review. Clin Rev Allergy Immunol 2015;49:100–51. 26445775 10.1007/s12016-015-8509-4

[4] Galvez-Sánchez CM, Duschek S, Reyes Del Paso GA. Psychological impact of fibromyalgia: current perspectives. Psychol Res Behav Manag 2019:117–27. 30858740 10.2147/PRBM.S178240 PMC6386210

[5] Verbunt JA, Pernot DH, Smeets RJ. Disability and quality of life in patients with fibromyalgia. Health Qual Life Outcomes 2008;6:1–8. 18211701 10.1186/1477-7525-6-8 PMC2265693

[6] Chakrabarty S, Zoorob RFibromyalgia. Am Fam Physician 2007;76:247–54. 17695569

[7] Baiardini I, Braido F, Giardini A, Majani G, Cacciola C, Rogaku A, Scordamaglia A, Canonica GW. Adherence to treatment: assessment of an unmet need in asthma. J Investig Allergol Clin Immunol 2006;16:218. 16889278

[8] Jankowska-Polańska B, Uchmanowicz I, Dudek K, Mazur G. Relationship between patients’ knowledge and medication adherence among patients with hypertension. Patient Prefer adherence 2016:2437–47. 27994443 10.2147/PPA.S117269 PMC5153315

[9] Sweileh WM, Zyoud SH, Abu Nab’a RJ, Deleq MI, Enaia MI, Nassar SM, Al-Jabi SW. Influence of patients’ disease knowledge and beliefs about medicines on medication adherence: findings from a cross-sectional survey among patients with type 2 diabetes mellitus in Palestine. BMC Public Health 2014;14:1–8. 24479638 10.1186/1471-2458-14-94 PMC3909379

[10] Arnold LM, Clauw DJ, Dunegan LJ, Turk DC. A framework for fibromyalgia management for primary care providers 2012;87:488–96. 10.1016/j.mayocp.2012.02.010 PMC3498162 22560527

[11] Louw A, Diener I, Butler DS, Puentedura EJ. The effect of neuroscience education on pain, disability, anxiety, and stress in chronic musculoskeletal pain. Arch Phys Med Rehabil 2011;92:2041–56. 22133255 10.1016/j.apmr.2011.07.198

[12] Louw A, Zimney K, Puentedura EJ, Diener I. The efficacy of pain neuroscience education on musculoskeletal pain: a systematic review of the literature. Physiother Theory Pract 2016;32:332–55. 27351541 10.1080/09593985.2016.1194646

[13] Moseley L. Unraveling the barriers to reconceptualization of the problem in chronic pain: the actual and perceived ability of patients and health professionals to understand the neurophysiology. J Pain 2003;4:184–9. 14622702 10.1016/s1526-5900(03)00488-7

[14] de Oliveira Lima L, Saragiotto BT, Costa LOP, Nogueira LC, Meziat-Filho N, Reis FJ. Self-guided web-based pain education for people with musculoskeletal pain: a systematic review and meta-analysis. Phys Ther 2021,101:pzab167. 34174081 10.1093/ptj/pzab167

[15] Mehta S, Peynenburg VA, Hadjistavropoulos HD. Internet-delivered cognitive behaviour therapy for chronic health conditions: a systematic review and meta-analysis. J Behav Med 2019;42:169–87. 30387008 10.1007/s10865-018-9984-x

[16] Mann EG, LeFort S, VanDenKerkhof EG. Self-management interventions for chronic pain. Pain Manag 2013;3:211–22. 24654764 10.2217/pmt.13.9

[17] Heapy AA, Stroud MW, Higgins DM, Sellinger JJ. Tailoring cognitive-behavioral therapy for chronic pain: A case example. J Clin Psychol 2006;62:1345–54. 16937347 10.1002/jclp.20314

[18] Furness PJ, Vogt K, Ashe S, Taylor S, Haywood-Small S, Lawson K. What causes fibromyalgia? An online survey of patient perspectives. Health Psychol Open 2018; 5:2055102918802683. 30275965 10.1177/2055102918802683 PMC6158621

[19] Harris PA, Taylor R, Minor BL, Elliott V, Fernandez M, O’Neal L, McLeod L, Delacqua G, Delacqua F, Kirby J. The REDCap consortium: building an international community of software platform partners. J Biomed Inform 2019;95:103208. 31078660 10.1016/j.jbi.2019.103208 PMC7254481

[20] Harris PA, Taylor R, Thielke R, Payne J, Gonzalez N, Conde JG. A metadata-driven methodology and workflow process for providing translational research informatics support. J Biomed Inform 2009;42:377–81. 18929686 10.1016/j.jbi.2008.08.010 PMC2700030

[21] Braun V, Clarke V. Using thematic analysis in psychology. Qualitative research in psychology 2006;3:77–101.

[22] SocioCultural Research Consultants L. Dedoose Version 9.0.107, cloud application for managing, analyzing, and presenting qualitative and mixed method research data;9.0.107.

[23] Geneen LJ, Martin DJ, Adams N, Clarke C, Dunbar M, Jones D, McNamee P, Schofield P, Smith BH. Effects of education to facilitate knowledge about chronic pain for adults: a systematic review with meta-analysis. Syst Rev 2015;4:1–21. 26428467 10.1186/s13643-015-0120-5 PMC4591560

[24] Arlinghaus KR, Johnston CA. Advocating for behavior change with education. Am J Lifestyle Med 2018;12:113–6. 30283247 10.1177/1559827617745479 PMC6124997

[25] Yavne Y, Amital D, Watad A, Tiosano S, Amital H. A systematic review of precipitating physical and psychological traumatic events in the development of fibromyalgia 2018;48:121–33. 10.1016/j.semarthrit.2017.12.011 29428291

[26] Wolfe F, Häuser W, Walitt BT, Katz RS, Rasker JJ, Russell AS. Fibromyalgia and physical trauma: the concepts we invent. J Rheumatol 2014;41:1737–45. 25086080 10.3899/jrheum.140268

[27] Allen KD, Coffman CJ, Golightly YM, Stechuchak KM, Keefe FJ. Daily pain variations among patients with hand, hip, and knee osteoarthritis. Osteoarthr Cartil 2009;17:1275–82. 10.1016/j.joca.2009.03.021 19410670

[28] Bartley EJ, Robinson ME, Staud R. Pain and fatigue variability patterns distinguish subgroups of fibromyalgia patients. J Pain 2018;19:372–81. 29253551 10.1016/j.jpain.2017.11.014 PMC5869098

[29] Harris RE, Williams DA, McLean SA, Sen A, Hufford M, Gendreau RM, Gracely RH, Clauw DJ. Characterization and consequences of pain variability in individuals with fibromyalgia. Arthritis Rheum 2005;52:3670–4. 16258905 10.1002/art.21407

[30] Prince A, Bernard AL, Edsall PA. A descriptive analysis of fibromyalgia from the patients’ perspective. J Musculoskelet Pain 2000;8:35–47.

[31] Hallberg LR, Bergman S. Minimizing the dysfunctional interplay between activity and recovery: A grounded theory on living with fibromyalgia. Int J Qual Stud Health Well-being 2011;6:7057. 10.3402/qhw.v6i2.7057 PMC3114951 21687553

[32] Bragazzi NL, Amital H, Adawi M, Brigo F, Watad S, Aljadeff G, Amital D, Watad A. What do people search online concerning the “elusive” fibromyalgia? Insights from a qualitative and quantitative analysis of Google Trends. Clin Rheumatol 2017;36:1873–8. 28462482 10.1007/s10067-017-3665-y

[33] Madden S, Sim J. Creating meaning in fibromyalgia syndrome. Soc Sci Med 2006;63:2962–73. 16949713 10.1016/j.socscimed.2006.06.020

[34] Nicola M, Correia H, Ditchburn G, Drummond P. Invalidation of chronic pain: a thematic analysis of pain narratives. Disabil Rehabil 2021;43:861–9. 31290347 10.1080/09638288.2019.1636888

[35] Kool MB, Van Middendorp H, Lumley MA, Schenk Y, Jacobs J, Bijlsma J, Geenen R. Lack of understanding in fibromyalgia and rheumatoid arthritis: the Illness Invalidation Inventory (3* I). Ann Rheum Dis 2010;69:1990–5. 20498203 10.1136/ard.2009.123224

[36] Slater H, Jordan JE, Chua J, Schütze R, Wark JD, Briggs AM. Young people’s experiences of persistent musculoskeletal pain, needs, gaps and perceptions about the role of digital technologies to support their co-care: a qualitative study. BMJ Open 2016;6:e014007. 10.1136/bmjopen-2016-014007 PMC5168607 27940635

[37] Lobo CP, Pfalzgraf AR, Giannetti V, Kanyongo G. Impact of invalidation and trust in physicians on health outcomes in fibromyalgia patients. Prim care Companion CNS Disord 2014:16:27355. 10.4088/PCC.14m01664 PMC4321014 25667809

[38] Eisenberger NI, Jarcho JM, Lieberman MD, Naliboff BD. An experimental study of shared sensitivity to physical pain and social rejection. Pain 2006;126:132–8. 16890354 10.1016/j.pain.2006.06.024

[39] Häuser W, Thieme K, Turk DC. Guidelines on the management of fibromyalgia syndrome–a systematic review. Eur J Pain 2010;14:5–10. 19264521 10.1016/j.ejpain.2009.01.006

[40] Macfarlane GJ, Kronisch C, Dean LE, Atzeni F, Häuser W. Fluß E1, Choy E, Kosek E, Amris K, Branco J. EULAR revised recommendations for the management of fibromyalgia. Ann Rheum Dis 2017;76:318–28. 27377815 10.1136/annrheumdis-2016-209724

